# Benefit of *N*-Acetylcysteine in Postoperative Hepatic Dysfunction: Case Report and Review of Literature

**DOI:** 10.1155/2019/4730381

**Published:** 2019-12-17

**Authors:** Deanna K. Bauerlein, Hashem N. Akbar, Erik C. von Rosenvinge, Nora D. Loughry, Preeti R. John

**Affiliations:** ^1^Veterans Affairs New Jersey Healthcare System, USA; ^2^Tufts University Medical Center, USA; ^3^Veterans Affairs Maryland Healthcare System, University of Maryland School of Medicine, USA; ^4^Emory University School of Medicine, USA

## Abstract

*N*-Acetylcysteine (NAC) is reported to have multiple clinical applications in addition to being the specific antidote for acetaminophen toxicity. NAC stimulates glutathione biosynthesis, promotes detoxification, and acts directly as a scavenger of free radicals. It is a powerful antioxidant and a potential treatment option for diseases characterized by the generation of free oxygen radicals. We present a case of postoperative hepatic dysfunction of multifactorial etiology in a patient with therapeutic acetaminophen levels, where hepatic function improved considerably following administration of intravenous NAC. This case suggests that NAC should be considered for treatment of acute liver dysfunction in the postoperative setting, even in the absence of elevated acetaminophen levels.

## 1. Introduction

*N*-Acetylcysteine (NAC) is the antidote for acetaminophen (APAP) toxicity and has been used as a beneficial drug treatment for liver disorders. Its use and benefits have been reported in multiple other conditions and is attributed to the fact that it increases levels of glutathione, the body's major antioxidant [1].

We present a patient who developed hepatic dysfunction of multifactorial etiology in the postoperative period, whose hepatic function improved soon after administration of intravenous (IV) NAC. The patient had hepatitis C virus (HCV), alcohol use disorder (AUD) with liver function tests suggestive of alcoholic hepatitis, and borderline malnutrition. He received oral APAP for postoperative analgesia. It is believed that these factors combined to cause the development of acute hepatic dysfunction postoperatively. Following NAC administration, hepatic function improved rapidly.

## 2. Case Description

A 58-year-old male underwent elective left lower lobe resection via video-assisted thoracoscopic surgery (VATS) for a 9 mm lung nodule suspicious for malignancy. The patient's past medical history included hypertension, gastroesophageal reflux disease, depression, anxiety, chronic hepatitis C, and AUD. Liver biopsy and Fibroscan had not been performed to assess for cirrhosis, preoperatively. His last admission was six months prior to surgery for alcohol withdrawal syndrome. The patient reported drinking 10 beers a day with his last drink on the day before surgery. He denied tobacco, but reported using marijuana. He had no known allergies or adverse drug reactions.

His baseline liver function tests (LFTs) before surgery were: AST 111 U/L, ALT 50 U/L, total bilirubin (TBili) 0.5 mg/dl, INR 1.11 (Table 1). AST/ALT ratio suggested alcoholic hepatitis. Nutritional status was marginal, with albumin 3.1 g/dL. Platelet count was 120,000 k/cmm. Outpatient medications included aripiprazole, sertraline, and a multivitamin.

There were no hypotensive episodes or other complications during VATS. Blood loss was less than 50 ml. He received general anesthesia with sevofluorane. There were no other concomitant medications given intraoperatively or postoperatively that could have contributed to deteriorating hepatic function.

The following analgesics were given to optimize pain control postoperatively: APAP 1 g orally every six hours for three days, oxycodone 5–10 mg orally every four hours as needed, and hydromorphone 0.5 mg IV every four hours as needed for breakthrough pain.

**Figure 1:**
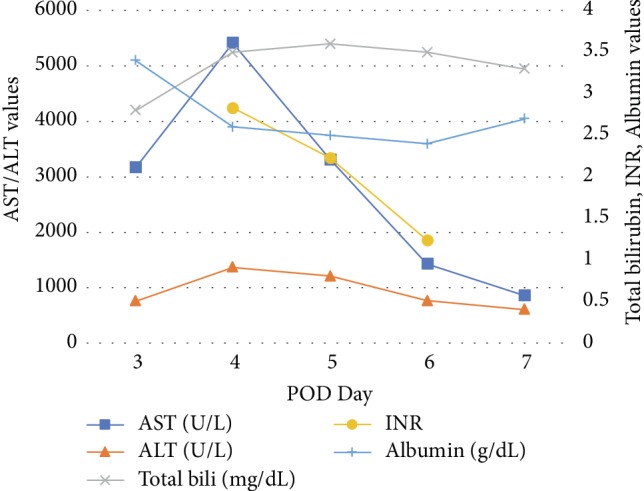
Selected laboratory trends.

On postoperative day 2 (POD 2), the patient remained hemodynamically stable. On POD 3, he developed nausea and discomfort in the right upper quadrant. LFTs were checked and noted to be elevated: AST 3169 U/L, ALT 758 U/L, TBili 2.8 mg/dL (Table 1, Figure 1). He had received a total of 9 g of APAP over a period of 3 days, with no more than 4 g over a 24-hour period. APAP was discontinued at this time due to LFT elevation and concern for iatrogenic hepatotoxicity.

On POD 4, LFTs peaked: AST 5417 U/L, ALT 1372 U/L, TBili 3.5 mg/dL; albumin 2.6 g/dL, INR 2.83, lactate 6.1 mmol/L; and glucose 58 mg/dl. Renal function and arterial blood gas remained within normal limits. Urine toxicology screen was negative. The patient reported right-sided abdominal pain and nausea, and had two episodes of emesis. He was noted to have scleral icterus and grade I encephalopathy. He had a syncopal episode secondary to hypoglycemia, which promptly responded to intravenous dextrose administration. A CT scan of the abdomen and pelvis was done, revealing fatty liver without portal vein thrombosis or other biliary tract abnormalities. Ischemic hepatitis (shock liver) was thought unlikely as the patient was never hypotensive.

The hepatology service was consulted and a diagnosis of acetaminophen-induced hepatotoxicity was suspected based on the clinical picture, laboratory values, and negative imaging. However, APAP level was within normal limits at 12.9 mg/L (normal therapeutic range 5–20 mg/L) [2]. Despite a nontoxic APAP level, NAC was recommended. On POD 4, he was treated with 3 weight-based doses of IV NAC: 9 g (150 mg/kg) over one hour, then 3 g (50 mg/kg) over four hours, followed by 6 g (100 mg/kg) over sixteen hours. A one-time dose of oral vitamin K 10 mg was also administered.

On POD 5, APAP level decreased to 0.5 mg/L and LFTs were noted to be declining. He was discharged on POD 7, at which time he was clinically stable and asymptomatic. LFTs continued to improve and the patient's renal function remained at baseline and within normal limits. One month after discharge, LFTs further decreased to: AST 427 U/L, ALT 82 U/L, TBili 2 mg/dL.

Throughout the patient's hospital stay, renal function remained at baseline (0.8–1.4 mg/dl).

## 3. Discussion

This case report describes the beneficial effect of IV NAC when used in the postoperative period, in a patient who developed hepatic dysfunction following oral APAP use for analgesia. Our patient had preexisting risk factors for acetaminophen-induced hepatoxicity including HCV, AUD with possible alcoholic hepatitis, and malnutrition. Expert opinion recommends limitation of APAP dosage to 2 g/day in patients with chronic or acute alcohol abuse [2]. However, this patient received APAP 4 g/day in adherence with current FDA recommendations for APAP use in adults. When measured, his APAP level was within normal limits at 12.9 mg/L. Therapeutic APAP blood concentration is ≤20 mg/L and toxic blood concentrations are typically in the range of 25–150 mg/L [3]. Although it is unlikely that APAP toxicity was the sole cause of his acute liver dysfunction, it likely contributed to it.

Treatment with NAC was recommended by hepatologists based on documented benefits of NAC use in liver dysfunction caused by various etiologies. Regardless of the etiology of acute liver dysfunction in this patient who underwent surgery, the use of IV NAC in the postoperative period was associated with rapidly improving liver function. The discontinuation of APAP also likely contributed to improving liver function.

There are only two case reports in the published literature that we are aware of, describing beneficial effects of IV NAC when used in the postoperative period in patients who developed hepatic dysfunction associated with APAP use. Both describe patients who developed hepatic dysfunction associated with intravenous (IV) APAP use. In this case report, we describe hepatic dysfunction associated with oral APAP use in the postoperative period.

The first case report describes a 36-year-old woman who underwent laparoscopic appendectomy and received a total of 16 g of IV APAP for pain control over 5 days in the peri-operative period. APAP level was 34 mg/L [4]. The patient had transaminase elevations that resolved with IV NAC administration. Malnutrition was the only known hepatotoxic risk factor in this patient [4].

The second report describes a 92-year-old female who received a total of 13 g of IV APAP (1 g every six hours) for pain control after laparoscopic reduction of internal hernias, which resulted in elevated LFTs. APAP level was within normal range. She had no history of hepatic disease or alcohol abuse. Her advanced age, postoperative status and likely catabolic state were thought to have caused susceptibility to acetaminophen-induced hepatotoxicity. LFTs improved with the administration of IV NAC [5].

NAC is a potential treatment option for both acute and chronic diseases characterized by the generation of free oxygen radicals [1]. There are several reports in the literature about benefits of NAC treatment for APAP toxicity [3, 6] and treatment of this condition constitutes its only FDA approved use in liver dysfunction.

NAC has antioxidant properties, which has been attributed to its role as a precursor of glutathione [7]. Oxidative stress is one of the pathogenetic mechanisms causing liver damage. It contributes to initiation and progression of steatosis, fibrosis, and hepatic dysfunction in a variety of liver disorders [8].

NAC stimulates glutathione biosynthesis by providing cysteine, which is involved in a rate-limiting step in glutathione synthesis. Glutathione is essential for the body's antioxidant defenses [9].

In addition to oxidative stress, nitrosative stress mechanisms are involved in the pathogenesis of chronic liver diseases. In the presence of reactive oxygen species, nitric oxide forms peroxynitrites which have deleterious effects [10]. Nitric oxide contributes to liver ischemia-reperfusion injury. Inhibition of inducible nitric oxide synthase (iNOS) shows beneficial effects and NAC modulates expression of iNOS in human hepatocytes stimulated by proinflammatory cytokines [10]. This may be the basis of beneficial effects of NAC in chronic liver diseases [10].

NAC has been used successfully for chronic liver disorders such as chronic hepatitis C [11, 12].

The American Association for the Study of Liver Diseases (AASLD) recommends NAC for all cases of acute liver failure, even if APAP is not the cause [13]. They suggest NAC administration if increasing aminotransferases are suggestive of liver injury, even if serum APAP level or amount of APAP ingested does not indicate APAP toxicity. This recommendation is based on the fact that NAC has the potential to improve liver function in nonacetaminophen-induced liver dysfunction [13].

A prospective, double-blind randomized controlled trial (RCT) concluded that IV NAC increased the transplant-free survival rate in patients who had nonacetaminophen-induced acute liver failure with coma grade I-II compared to placebo [14].

Another prospective, double-blind RCT showed that IV NAC improved hepatic function as shown by decreasing ALT and bilirubin values (but not AST, INR, or creatinine) and decreasing transplant rates in patients with nonacetaminophen-induced acute liver failure with coma grade I-II compared to placebo [15].

A third prospective, RCT demonstrated that IV NAC decreases mortality and hospital length of stay compared to placebo in patients with nonacetaminophen-induced acute liver failure with coma grade I–IV [16].

Furthermore, NAC may play a role in preventing postoperative complications. One study on the prevention of acute renal failure after cardiac surgery showed marked reduction in renal failure in a subgroup of patients placed on cardiopulmonary bypass, who received NAC [17]. Another small study of twenty-two patients concluded that NAC may be useful in preventing pulmonary complications in patients undergoing esophagectomy for cancer [18].

Multiple other benefits of NAC supplementation in various disorders have been supported by varying levels of scientific evidence. These include treatment of pulmonary conditions (chronic obstructive pulmonary disease, chronic bronchitis, asthma, idiopathic pulmonary fibrosis, influenza virus, renal conditions (contrast induced nephropathy prevention), and treatment of infertility in patients with polycystic ovarian syndrome [1]. There have also been reports about the benefit of NAC in some psychiatric disorders such as schizophrenia and bipolar disorder [9].

With regard to drug safety, NAC is relatively safe. In its oral form, mild gastrointestinal symptoms are the most common side effects [9]. When given intravenously at high doses (>3 g/day), anaphylactoid reactions can occur.

## 4. Conclusion

Intravenous NAC was beneficial in this patient who developed acute hepatic dysfunction in the postoperative period. Given its favorable risk/benefit ratio in both acute and chronic hepatic dysfunction as reflected in multiple published reports, the use of intravenous NAC should be considered in postoperative patients with worsening hepatic dysfunction of multifactorial etiology.

## Figures and Tables

**Table 1:** Pertinent laboratory values.

Labs (reference range)	Prior to surgery	POD 1	POD 2	POD 3	POD 4	POD 5	POD 6	POD 7
AST (0–37 U/L)	111			3169	5417	3318	1432	863
ALT (17–63 U/L)	50			758	1372	1209	766	607
Total bilirubin (0–1.3 mg/dl)	0.5			2.8	3.5	3.6	3.5	3.3
INR (0.83–1.18)	1.11				2.83	2.23	1.24	1.24
Alk phos (38–126 U/L)				110	119	104	137	215
Albumin (3.5–4.8 g/dl)				3.4	2.6	2.5	2.4	2.7
BUN (6–20 mg/dl)	4	3	6	9	10	8	5	2
Cr (0.9–1.3 mg/dl)	1.36	0.86	1.03	1.08	1.28	0.94	0.79	0.78
Glucose (70–105 mg/dl)	79	105	82	80	58	112	95	99
NH_3_ (9–35 mcMol/l)						52		
Lactate (0.5–2 mmol/L)					6.1	3.1		

## References

[1] Millea P. J. (2009). *N*-Acetylcysteine: multiple clinical applications. *American Family Physician*.

[2] Chandok N., Watt K. D. S. (2010). Pain management in the cirrhotic patient. *Mayo Clinic Proceedings*.

[3] Heard K. J. (2008). Acetylcysteine for acetaminophen poisoning. *The New England Journal of Medicine*.

[4] Lee P. J., Shen M., Wang S. (2015). Possible hepatoxicty associated with intravenous acetaminophen in a 36-year-old female patient. *P&T*.

[5] Seifert S. A., Kovnat D., Anderson V. E., Green J. L., Dart R. C., Heard K. J. (2016). Acute hepatoxicity associated with therapeutic doses of intravenous acetaminophen. *Clinical Toxicology*.

[6] Green J. L., Heard K. J., Reynolds K. M., Albert D. (2013). Oral and intravenous acetylcysteine for treatment of acetaminophen toxicity: a systematic review and meta-analysis. *The Western Journal of Emergency Medicine*.

[7] Mokhtari V., Afsharian P., Shahhoseini M., Kalantar S. M., Moini A. (2017). A review on various uses of *N*-acetylcysteine. *Cell Journal*.

[8] Medina J., Moreno-Otero R. (2005). Pathophysiological basis for antioxidant therapy in chronic liver disease. *Drugs*.

[9] Dodd S., Dean O., Copolov D. L., Malhi G. S., Berk M. (2008). *N*-acetylcysteine for antioxidant therapy: pharmacology and clinical utility. *Expert Opinion on Biological Therapy*.

[10] Moreno-Otero R. (2010). Hepatoprotective effects of antioxidants in chronic hepatitis C. *World Journal of Gastroenterology*.

[11] Nabi T., Nabi S., Rafiq N., Shah A. (2017). Role of *N*-acetylcysteine treatment in nonacetaminophen-induced acute liver failure: a prospective study. *Saudi Journal of Gastroenterology*.

[12] Melhem A., Stern M., Shibolet O. (2005). Treatment of CHC virus infection via antioxidants: results of a phase I clinical trial. *Journal of Clinical Gastroenterology*.

[13] Lee W. M., Larson A. M., Stravitz R. T. (2011). AASLD position paper: the management of acute liver failure: update 2011. https://www.aasld.org/sites/default/files/2019-06/AcuteLiverFailureUpdate201journalformat1.pdf.

[14] Lee W. M., Hynan L. S., Rossaro L. (2009). Intravenous *N*-acetylcysteine improves transplant-free survival in early stage nonacetaminophen acute liver failure. *Gastroenterology*.

[15] Singh S., Hynan L. S., Lee W. M. (2013). Improvements in hepatic biomarkers are associated with clinical benefit of intravenous *N*-acetylcysteine in early stage nonacetaminophen acute liver failure. *Digestive Diseases and Sciences*.

[16] Glantzounis G. K., Salacinski H. J., Yang W., Davidson B. R., Seifalian A. M. (2005). The contemporary role of antioxidant therapy in attenuating liver ischemia reperfusion injury: a review. *Liver Transplantation*.

[17] Sisillo E., Ceriani R., Bortone F. (2008). NAC for prevention of acute renal failure in patients with chronic renal insufficiency undergoing cardiac surgery: a prospective, randomized clinical trial. *Critical Care Medicine*.

[18] Zingg U., Hofer C., Seifert K., Metzger B. U., Zollinger A. (2007). High dose NAC to prevent pulmonary complications in partial or total transthoracic esophagectomy: results of a prospective observational study. *Diseases of the Esophagus*.

